# Systematic Literature Review of the Take-Home Route of Pesticide Exposure via Biomonitoring and Environmental Monitoring

**DOI:** 10.3390/ijerph16122177

**Published:** 2019-06-19

**Authors:** Nicolas López-Gálvez, Rietta Wagoner, Lesliam Quirós-Alcalá, Yoshira Ornelas Van Horne, Melissa Furlong, El'gin Avila, Paloma Beamer

**Affiliations:** 1 Department of Community, Environment & Policy, Mel and Enid Zuckerman College of Public Health, University of Arizona, Tucson, AZ 85724, USA; rwagoner@email.arizona.edu (R.W.); yornelas@email.arizona.edu (Y.O.V.H.); mfurlong@email.arizona.edu (M.F.); elginavila@email.arizona.edu (E.A.); pbeamer@email.arizona.edu (P.B.); 2 Department of Environmental Health & Engineering, Johns Hopkins University, Baltimore, MD 21205, USA; lalcala1@jhu.edu; 3 Maryland Institute of Applied Environmental Health, University of Maryland, MD 20740, USA

**Keywords:** take-home pathway, pesticide exposure, farmworkers, agricultural, para-occupational, rural, biomarkers, residues

## Abstract

Background: Exposure to pesticides via take-home can be an important pathway for farmworkers’ families. Objective: The aim of this review was to summarize and analyze the literature published during the last decade of exposure to pesticides via take-home pathway in farmworkers’ families. Methods: We conducted a systematic review to identify peer-reviewed articles of interest; only articles related to take-home pathway that included some sort of pesticide monitoring were considered for inclusion. Systematic reviews, literature reviews, and meta-analyses were excluded, resulting in a total of 39 articles elected for analysis. The articles were summarized based on the location of the study, population (sample size), pesticide analyzed, and type of sample. Results: The majority of the reviewed studies were conducted in the U.S., but there seems to be an increase in literature on pesticide take-home pathway in developing countries. Most of the articles provided evidence that farmworkers’ families are exposed to pesticides at higher levels than non-farmworkers’ families. The levels may depend on several factors such as seasonality, parental occupation, cohabitation with a farmworker, behavior at work/home, age, and gender. Community-based interventions disrupting the take-home pathway seem to be effective at reducing pesticide exposure. Discussion/Conclusion: The take-home pathway is an important contributor to overall residential exposures, but other pathways such as pesticide drift, indoor-residential applications, and dietary intake need to be considered. A more comprehensive exposure assessment approach is necessary to better understand exposures to pesticides.

## 1. Introduction

Worldwide, it is estimated that approximately 6 billion pounds of pesticides are used each year to kill or repel pests, including insects, plant life, and fungi [1]. A wealth of literature exists regarding human exposure to pesticides for agricultural workers, non-agricultural workers, and children alike. Multiple epidemiology studies have investigated the relation between exposure to pesticides and a variety of health effects such as cancer, asthma, birth defects, Parkinson’s disease, and diabetes [2]. These include exposures via occupational, para-occupational, residential, environmental, and dietary pathways. Animal toxicity studies also contribute to the body of knowledge concerning chronic exposures and effects [3]. Similarly, the acute effects of pesticides have been well documented in occupational and animal studies. Acute symptoms range from dizziness, irritation of the skin, eyes, or respiratory tract to neurotoxicity, extreme weakness, and even death [4]. Varying regulatory standards and recommendations exist throughout the world in order to reduce human exposure to pesticides via air, soil, water, and agricultural commodities [5]. 

Increased evidence exists for the “take-home” exposure pathway for pesticides. The take-home, or “para-occupational”, exposure pathway arises from the transfer of pesticide residues from the workplace to the household environment via agricultural workers’ clothes, skin, vehicles, and shoes [6]. Once in the home, pesticides can enter the bodies of household occupants through inhalation, ingestion, dermal contact, or some combination of these three [7]. Compared to adults, children’s exposure to pesticides in the home is of special concern because children spend an increased amount of time on floors where pesticide residues may settle, frequently engage in hand-to-mouth activity and mouthing of objects that can host pesticide residues, have increased inhalation rates, and consume more drinks and food items than adults do per kilogram of bodyweight [7,8,9,10]. To better understand the contribution of the take-home pathway to individuals’ exposure to pesticides, Coronado et al. (2011) conducted a literature review by summarizing articles published from 1990 to April 2008. In addition, two other literature reviews have been done on similar topics: Hyland and Laribi (2017) conducted a systematic review on take-home pesticide exposure pathway in children living in agricultural areas, while Deziel et al. (2015) identified 19 articles published from 1995 to 2013 regarding paraoccupational pesticide exposure in women living in North American agricultural areas [6,11,12].

The authors of the aforementioned literature reviews evaluated articles that assessed pesticide dust concentrations in houses and vehicles as well blood/urinary metabolite concentrations of farmworkers, farmworker children, and farmworker spouses. The authors illustrated a substantial amount of evidence suggesting that the take-home pathway of pesticide exposure is an important route by which household occupants are exposed to pesticides. To update this body of knowledge and expand upon this topic, this article provides an updated summary of the literature on the pesticide take-home pathway by systematically analyzing the available articles published from April 2008 to September 2018. This article reviews studies from North America, South America, Asia, Europe, and Australia, that included biomonitoring or environmental monitoring of farmworkers, farmworker children, and farmworkers’ spouses. Biological monitoring of pesticides included urine and/or blood. Environmental monitoring of pesticides included dust levels in the homes and vehicles of agricultural workers. This systematic literature review summarizes important information of the exposure factors that may contribute to the pesticide take-home pathway by comparison of pesticide residue in dust and/or pesticide biomarkers in agricultural communities vs. non-agricultural communities, seasonality, household and individual characteristics, and interventions aimed at reducing exposure. The information gained from this review can highlight areas for future studies and help develop improved interventions and regulations to reduce exposure to pesticides via the take-home pathway. 

## 2. Methods

**Figure 1:**
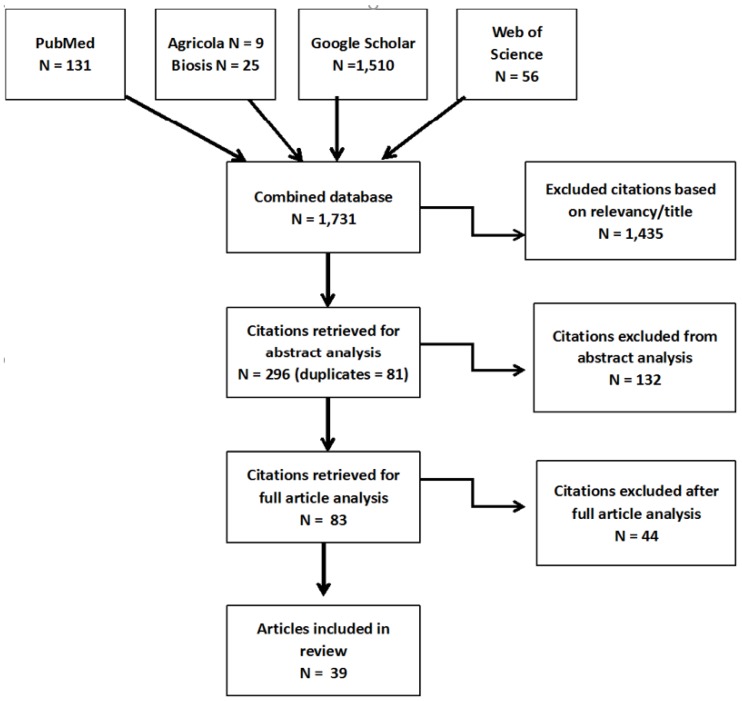
Flowchart of article selection.

A systematic approach was conducted by following the PRISMA guidance protocol (http://www.prisma-statement.org/). To identify relevant studies, we used Google Scholar, Agricola, Biosis, PubMed, and Web of Science. This search was focused only on peer-reviewed journal articles that were published between April 2008 to 30 September 2018. The studies were selected using the following search terms to specify the location and activity: ‘agricultural’, ‘agriculture’, ‘rural’, ‘farm’, ‘applicator’, or ‘farmworker’; to specify the exposure: ‘pesticide’, ‘pesticide drift’, ‘pesticide exposure’, or ‘pesticide measurement’; to specify the pathway: ‘take-home’, ‘para-occupational’, ‘indoor’, ‘house’, ‘home’, ‘residential’, or ‘housing’; and to specify the type of samples or media: ‘dust’, ‘urine’, ‘urinary’, or ‘blood’. An example of the article search using PubMed: (((“agricultural”[Title/Abstract] OR “agriculture”[Title/Abstract] OR “rural”[Title/Abstract] OR “farm”[Title/Abstract] OR “applicator”[Title/Abstract] OR “farmworker”[Title/Abstract]) AND (pesticide*[All Fields] OR “pesticide drift” [All Fields] OR “pesticide exposure” [All Fields] OR “pesticide measurement” [All Fields]) AND (take-home *[All Fields] OR “indoor”[All Fields] OR “house”[All Fields] OR “home”[All Fields] OR “residential” [All Fields] OR “housing”[All Fields]) AND (“dust”[All Fields] OR“urine”[All Fields] OR “urinary”[All Fields] OR “blood”[All Fields])) AND (“2008/04/01”[PDat] : “2018/09/30”[PDat])). The articles were coded by five researchers: Teams of two were assigned to each database. Each researcher independently identified and reviewed the articles from their assigned database, either PubMed, Web of Science, Google Scholar, Agricola, or Biosis. One researcher was also responsible for organizing all of the articles and assisting with the elimination of duplicates. The search provided a total of 1731 articles. Only studies that were available in English, focused on agricultural workers or farmworkers families, and met the inclusion criteria related to the topic of take-home pathway were included in this analysis. Articles were excluded if not relevant to the take-home pathway topic. Methodology, meta-analysis, and review articles were not included in this analysis. It is important to mention that no geographical limitation was applied to the analysis. We excluded 1435 due to their lack of relevance, 132 based on review of the abstract, and 44 based on the full article review (Figure 1). Finally, 39 articles were identified for final inclusion. The studies were grouped by the type of samples collected to evaluate the take-home exposure pathway and included either biological or environmental samples. The articles were summarized using the following information: author (date), location of the study, population (sample size), pesticide analyzed, type of sample, and primary conclusions, as presented in Table 1, Table 2, Table 3 and Table 4.

## 3. Results

The selected 39 articles provide relevant information for assessing the pesticide take-home pathway and were grouped and summarized based on measurements of pesticides and/or metabolites in biological and environmental samples as shown in Table 1, Table 2, Table 3 and Table 4. This systematic literature review incorporated articles that included many different study designs, assessed different classes of pesticides, and utilized a large range of methodologies to assess pesticide exposure. Summarized in Table 1 are the studies that analyzed pesticide biomarkers in participants’ blood (n = 5, 13%), the studies that analyzed urinary pesticide metabolites only (n = 12, 31%) are shown in Table 2, the studies that focused on urinary pesticide metabolites as well as pesticide concentrations in dust (n = 10, 25%) are summarized in Table 3, and finally, the studies that analyzed pesticide dust and/or air concentrations (n = 12, 31%) are presented in Table 4. Overall, the majority of studies were conducted in the U.S. (n = 25, 64%) followed by Asia (n = 9, 23%), Europe (n = 2, 5%), Latin America (n = 2, 5%), and Australia (n = 1, 3%). Within the U.S, most of the reviewed studies were conducted in the Northwest (n = 12, 31%) followed by California (n = 10, 26%), North Carolina (n = 2, 5%), and Iowa (n = 1, 3%). Organophosphates (OPs) and their metabolites were the main pesticides analyzed in these studies (n = 32, 82%), followed by pyrethroids (n = 11, 28%), and organochlorines (n = 2, 5%). Six studies (15%) analyzed herbicides and fungicides, including one study (3%) on glyphosate. 

### 3.1. Evidence of Take-Home Pathway

The majority of the reviewed articles (n = 34, 87%) provided strong evidence that supports the take-home pathway. Several of the articles identified in this literature review showed that rural populations and farmworkers have higher concentrations of pesticides in either biological samples or house dust than their non-rural or non-farmworker counterparts [13,14,15,16]. In addition, people living in rural areas or those living with farmworkers are reported to have more adverse health effects, including cholinesterase inhibition and genotoxicity [17]. Of course, this evidence alone is insufficient to pinpoint the take-home pathway as the primary exposure route since the associations may be explained partially or entirely by exposure to pesticide drift, occupational exposure, or differential dietary and residential use patterns. In addition, 4 out of the 39 articles (10%) reported no evidence to support the take-home pathway [18,19,20,21].

Evidence supporting the take-home pathway includes children’s co-habitation with flower workers in Ecuador, along with the number of “bad practices” (wearing working clothes in home, not washing hands after work, etc.) by the flower workers. These factors were associated with lower systolic blood pressure among children in the flower-worker households [22]. This relationship held even after accounting for distance to nearest field, strengthening the evidence that this health outcome was independent of agricultural drift. In another study that assessed pesticide exposure via occupational take-home pathways against distance to field, Gunier and colleagues (2011) showed that although distance to field was one of the more important factors in predicting pesticide concentration in house dust in California, residing with an agricultural worker did explain a small, but significant, additional amount of variance [23]. Interestingly, in a study comparing 54 farmworkers’ and 54 non-farmworkers’ households in Thailand that were all situated within 150 m of a chili-farm, the authors reported no significant differences in OP air or dust concentrations between farmworker and non-farmworker homes [24]. In addition, Quiros-Alcala et al. (2011) found that dust concentrations for some pesticides, including OPs metabolites such as Dialkylphosphates (DAP), were higher in farmworkers’ homes compared to urban homes [25]. Furthermore, pesticides used in agricultural settings were detected in farmworker children’s homes and not in urban children’s homes. These findings imply that pesticide exposures from the take-home pathway as well as the drift pathway should be considered in research studies among populations near agricultural fields [24]. 

If the take-home pathway is an important route, then pesticide safety practices and behaviors should similarly result in reduced pesticide concentrations at home. Indeed, farmworkers that have access to or engage in practices that increase pesticide safety, including availability of laundry facilities, storing work boots at work instead of at home, frequent washing of hands before leaving the workplace, carpooling with other workers, parking car away from fields while at work, having ever received pesticide training, and recent receipt of pesticide safety training have been linked with lower household pesticide dust concentrations [26]. 

### 3.2. The Use of Biomarkers in Mothers/Spouses to Investigate the Take-Home Pesticide Exposure Pathway

To further examine the potential contribution of the take-home exposure pathway, five research studies (13%) measured pesticide biomarker concentrations in family members of farmworkers, including spouses or partners of farmworkers [13,15,27,28,29]. Results from these studies are inconsistent with regards to reported associations between pesticide exposures and potential take-home exposure related factors. These inconsistencies may be due to differences in study design, geographic study location, types of biomarkers of exposure (e.g., urine vs. blood; first morning urine voids vs. random spot urine samples; measurement of parent compounds vs. metabolites), analytical laboratory methods used to measure metabolites and/or precursor pesticides in biospecimens, and information collected via questionnaire related to take-home exposure factors. 

For example, a study by Huen et al. (2012) measured urinary and blood biomarker concentrations among mothers and newborns living in an agricultural community in a longitudinal birth cohort, Center for the Health Assessment of Mothers and Children of Salinas (CHAMACOS) in Salinas, California [15]. The authors reported null associations between detection of OP biomarkers in blood collected shortly before delivery or in umbilical cord blood and exposure determinants potentially related to the take-home exposure pathway (e.g., maternal occupation, living with farmworkers). Interestingly, 40% of mothers were farmworkers themselves and over 80% lived with one or more farmworkers. However, this null association may have been due to the matrix selected to assess OP pesticide exposures. OP pesticides are rapidly metabolized in the human body, leading to inherently low concentrations and subsequent low detection in blood [30]. In fact, few mothers and newborns in the Huen et al. (2012) study had quantifiable concentrations of OPs in blood. While concentrations of OP metabolites were measured in urine samples in the same study, the authors did not expand on the association between urinary levels and take-home exposure related factors [15]. 

In a study from an agricultural region of Thailand, mothers who lived with individuals that worked in agricultural fields had significantly higher levels of pesticides in their urine both during and after pregnancy than mothers who did not reside with agricultural workers [31]. Significant associations were identified between mothers’ urinary biomarker levels and living in proximity to farmlands amongst the mothers performing agricultural activities, such as applying pesticides in the fields. Other studies have also examined pesticide biomarker concentrations during the postnatal period. One study conducted in Pakistan did not observe any statistically significant differences in mean serum concentrations for several pesticide biomarkers (hexachlorobenzene, pentachlorophenol, p,p’-DDE, p,p’-DDT), between 17 mothers living in a rural area and 17 mothers living in an urban area. However, authors noted that although agriculture was the main activity in the rural study area, heavy use of pesticides was not common practice in the region **[13]**. 

Trunnelle et al. (2014) measured urinary pyrethroid metabolite concentrations among a subset of mothers (n = 105) and children (n = 103) aged 2–8 years from the Mexican Immigration to California: Agricultural Safety and Acculturation (MICASA) cohort in Mendota, CA [28]. The authors noted that biomarker concentrations for 3-phenoxybenzoic acid (3-PBA), a non-specific metabolite for several pyrethroid insecticides, were approximately 10 times higher in the cohort than those reported in women from the general U.S. population and also higher than in women participating in other farmworker studies in North Carolina [32]. It is not clear if findings were due to residence near an agricultural community with higher pesticide use or unique behaviors that led to higher para-occupational exposures. Further evidence for the take-home pathway comes from another study conducted in Thailand by Hanchenlaksh et al. (2011) where researchers found that the main determinant of urinary OP metabolites among farmworker spouses was whether the farmworkers had showered immediately after work or not [33]. Spouses whose partners did not shower had almost 15 times higher urinary concentrations of OP metabolites than the spouses of farmworkers who reported showering immediately after work. It is important to mention that the authors of these studies attribute their findings to not only the take-home pathway, but to additional burden of precursor pesticide exposure from factors such as living and/or working in an agricultural area, providing further evidence for the importance of investigating residential proximity, pesticide spray drift, and community exposures simultaneously.

### 3.3. The Use of Biomarkers in Children to Evaluate Take-Home Pesticide Exposure Pathway

A total of 25 (64%) studies utilized some type of biomarkers in children to determine exposure via the take-home pathway. There were 19 (49%) studies that investigated pesticide biomarker concentrations in children’s urine [9,14,17,18,21,27,28,29,31,33,34,35,36,37,38,39,40,41,42]. The remaining six studies investigated pesticide biomarkers in blood or household dust. Many of the studies reviewed reported higher urine metabolite concentrations in children of agricultural workers compared to children of non-agricultural workers. However, results from these studies are not conclusive. 

In a study conducted in Thailand, Panuwet et al. (2009) enrolled 207 children of farmers, laborers, and government employees to evaluate urinary pesticide metabolite concentration. The authors reported that the children of farmers had significantly higher concentrations of pyrethroid metabolites (t-DCCA and 3-PBA) and herbicide metabolites (2,4-D) in urine compared to the children of non-agricultural workers [38]. Similarly, levels of chlorpyrifos urine metabolite 3,5,6-trichloro-2-pyridinol (TCPy) were significantly lower in children of farmers than children of laborers [38]. Another study in Malaysia of 180 children found that most of the children with detectable pesticide urinary metabolites reported having fathers who worked in agricultural areas [17]. Also, in a study conducted in a predominantly agricultural province of Southeast China, it was reported that 12-month-old infants who had a father working in agriculture had almost two-fold higher urinary concentrations of pyrethroid metabolites compared to infants whose fathers were unemployed or reported having another occupation [29]. These results are supported by a study in Australia in which the high levels of urinary DEP in children were significantly associated with parental occupational exposure related to agricultural activities [14]. 

The findings from these articles suggest that having a family member working in agriculture may increase risk for exposure to pesticides in the household, unless the households are already in heavily contaminated regions. Thus, the cleaning and washing of pesticides from the worker before returning home is likely to reduce family members’ exposure to pesticide residues. For example, DAP levels in children’s urine was 22-fold higher if the parents washed themselves at home compared to if they washed themselves at work prior to returning home [33]. Similarly, one case study assessed glyphosate levels in a farmer and his family because two of his three children had birth defects. The authors reported that one of the children had detectable urinary concentrations two days after the father applied glyphosate to his farm, but not before [27]. However, the herbicide was not detected in the mother or any other children. Detection in the child may have been due to take-home exposure related factors such as from direct contact with the father who did not shower after spraying but only changed his clothes and washed his hands. Proximity is not likely since the farm was more than 1500 m from the home [27]. This suggests that the take-home pathway is an important factor to consider when assessing the exposure to pesticides and that certain household behaviors may reduce the risks from this pathway. 

Several of the reviewed studies (5, 13%) opted to assess pesticide exposure by utilizing blood measurements in children. Huen et al. (2012) measured OP metabolites in blood and found an association between mothers’ and children’s blood levels of OP metabolites [15]. It is also common to monitor blood cholinesterase levels to evaluate the extent of OP exposure. Only two of the reviewed studies evaluated blood cholinesterase activity as a tool to assess the take-home pathway of OPs among children residing near farms and cohabitating with farmers/farm workers [22,43]. One study included 277 Ecuadorian children living in a rural area and found that children living closer to a flower plantation and/or cohabitating with flower workers had significant lower acetylcholinesterase (AChE) activity [43]. Two studies by Ali et al. (2013) and Gonzalez-Alzaga et al. (2018) evaluated organochlorine (OC) concentrations in children’s blood serum [13,19]. Although Ali et al. (2013) found a significant correlation between the OC metabolite concentrations of mothers and children living in rural areas, Gonzalez-Alzaga et al. (2018) found no significant associations for the pesticide concentration in serum and father/mother occupational activity. However, it is important to mention that the Gonzales-Alzaga et al. (2018) study was recently conducted in Spain where the use of OCs have been banned. The levels observed in this study may have come from other pathways, such as food intake, due to the known persistency of OCs in the environment. 

### 3.4. Pesticide Residues in Household and Vehicle Dust

As presented in Table 4, 12 (31%) studies investigated the take-home pathway by measuring pesticides or pesticide metabolites in dust collected from agricultural workers’ homes and vehicles. Supporting the take-home pathway, two recent studies conducted in the U.S. Northwest found that pesticide residues in house dust may increase in relation to the number of residents working in agriculture [44,45]. In another study, Thompson et al. (2008) collected dust from 210 farmworker homes and 204 farmworker vehicles [21]. In house dust, several OPs were measured: azinphos-methyl concentrations ranged from <LOD to 10,487 ng/g, phosmet from <LOD to 13,804 ng/g, and malathion from <LOD to 1030 ng/g. OPs were also measured in vehicle dust: azinphos-methyl ranged from <LOD to 38,300 ng/g, phosmet from <LOD to 10,600 ng/g, and malathion <LOD to 21,504 ng/g. The levels of pesticides in the house dust were generally lower than the levels found in vehicle dust. This is an important pathway to consider as measuring pesticides only in the home may underestimate farmworkers’ and farmworker family exposure [21]. In a study by Trunnelle et al. (2013), different pyrethroid concentrations were measured in the house dust of 55 farmworker family homes in Mendota, CA. Cis-and trans-permethrin median concentrations were 244 and 172 ng/g dust, respectively [46]. Cypermethrin in the homes had a median concentration of 186 ng/g dust. While these concentrations are not particularly different than what has been previously been measured, this study contributes further evidence that farmworker homes face exposures to pyrethroid pesticides [46]. Fenske et al. (2013) also collected dust from commuter vehicles and farmworker homes [26]. Dust was analyzed for four OP pesticides (azinphos-methyl, phosmet, chlorpyrifos, malathion). While phosmet, malathion, and chlorpyrifos concentrations in vehicle dust were higher than in house dust, only azinphos-methyl dust concentrations were significantly higher [26]. Findings from both Thompson et al. (2008) and Fenske et al. (2013) support the hypothesis that the vehicle dust pathway is an important contributor to the overall take-home pathway, as OPs were generally higher in vehicle than house dust. Quirós-Alcalá et al. (2011) collected up to two dust samples from 13 urban homes in Oakland, California and 15 farmworker homes in Salinas, California. Median concentrations of diazinon, chlorpyrifos, per- methrins, allethrin, and chlorthal-dimethyl were higher in farmworker homes compared to urban homes [25]. Similar findings were reported by Thompson et al. (2014), where farmworker homes consistently had higher OP residue concentrations in house dust and vehicle dust compared to non-farmworker homes [42]. A significant correlation of 0.47 (*p* = 0.005) was reported between total dimethyl OP concentrations in vehicle dust and total dimethyl metabolite concentration in urine for adult farmworkers. For non-farmworker adults, a non-significant correlation of 0.19 (*p* = 0.10) between vehicle dust and urine was reported. These studies confirm that OPs and pyrethroids are frequently detected in house dust of farmworker homes. Only two looked at the differences between farmworkers and non-farmworkers, with both studies finding higher levels in farmworker than non-farmworker homes [25,42]. This reveals support for the occupational take home pathway for farmworkers, as OPs and pyrethroids are higher in house dust and vehicle dust than non-farmworker homes and vehicles. 

### 3.5. Household Characteristics

A few of the reviewed studies (9, 23%) investigated the relationship between household characteristics, both participant-reported and observations by researchers, to urinary pesticide metabolite levels of household occupants or to household pesticide dust levels [28,29,47,48,49,50,51,52,53]. In general, self-reported household characteristics were collected during in-home interviews with an adult either employed in agriculture or partnering/cohabitating with an agricultural worker. For studies involving self-reported household characteristics, urine was collected from adults, children, and/or infants in the household and analyzed for various pesticide metabolites. Self-reported home disrepair, which included water damage, rotting wood, peeling paint, and other visual indicators; self-reported spraying of residential pesticides indoors and outdoors; and the self-reported presence of cats and dogs allowed inside the home positively predicted urinary pesticide metabolite levels [28,29,49]. Interestingly, house cleanliness, vacuuming the carpet at least once per week, and wet mopping the floor on a daily basis were self-reported household behaviors that were investigated but did not have strong associations with urinary pesticide metabolite levels [48,49,51,53].

Observational household characteristics made by the research team or trained community assistants were completed in conjunction with the collection of urine or household dust [28,47,50,52]. Urine was collected from adults and children in the household and analyzed for various pesticide metabolites, including diazinon, pyrethroids, cyfluthrin, chlorpyrifos, and permethrins [28,52]. Household dust was collected by either high-volume surface samplers or wipes and analyzed for multiple pesticides, including organophosphates, pyrethroids, herbicides, and fungicides [47,50]. Observational indoor housing conditions, including presence of rodent/insects themselves or their feces, stains on floors, and peeling paint positively predicted urinary pesticide levels for children and dust concentrations in the home [28,50]. In regard to pests, the presence of roaches in the household was associated with increased levels of pesticides in dust and urinary pesticide metabolites [47,52]. Overcrowding/increased housing density were also associated with increased pesticide levels in household dust [47,50]. Conversely, Harnly et al. (2009) reported lower concentrations of organophosphates in the dust of homes that had an air conditioner [50]. Additionally, an observational study conducted in North Carolina reported that lower concentrations of pesticides on wipe samples were associated with the following household characteristics derived from the North Carolina Department of Labor Introduction to Migrant Housing Inspections housing assessment: Presence of workers with H-2A visas, posted certificate of inspection, absence of barracks, no floor violations, and no weather protection violations [47]. These latter household factors are additional variables that could confound studies attempting to understand the relative importance of the take-home exposure pathway.

### 3.6. Individuals’ Behaviors

A small number of the studies (5.13%) considered the associations between individual behaviors within the home and household dust or urinary pesticide metabolite levels [18,26,49,50,51,52]. Changing out of work clothes and work shoes inside of the home were the two primary behaviors that were associated with increased presence of dust or urinary pesticide metabolites [49,50,51,52]. Additionally, a study that analyzed pesticide residues in dust and urine metabolites of dimethylthiophosphate (DMTP) in 95 adult orchard workers and 94 of their children found that use of hand sanitizer was associated with increased urinary DMTP concentrations compared to those who did not use hand sanitizer [18]. This study suggests that a common home/work practice, use of hand sanitizers, that has been promoted to protect and reduce microbial spread in human environments may possibly increase absorption of pesticides. 

### 3.7. Interventions Designed to Reduce the Pesticide Take-Home Exposure Pathway

A few studies (5.13%) describe interventions at the workplace and/or community levels in order to reduce the amount of pesticides transported via the take-home exposure pathway [20,21,26,37,54]. Fenske et al. (2013) addressed potential intervention strategies at the workplace level. To reduce pesticide exposure levels, the authors recommended isolating worker clothing and boots by providing workers with space to store and/or clean contaminated clothing and boots as well as encouraging regular vacuuming of commuter vehicles [26]. Thompson et al. (2008) designed an extensive intervention that involved input from a diverse community advisory board and included various activities at the community level (e.g., health fairs, block parties), organizational level (e.g., elementary schools, churches, farm worker clinics, worksites, etc.), small group level (e.g., small home health parties), and individual level (e.g., door-to-door conversations) [21]. Activities were carried out by study staff and community health workers, known as *promotoras* in Spanish. Communities were randomly assigned to either the intervention or control group. Urinary metabolite and dust concentrations from the intervention group were not significantly different from the control group [21]. Plausible explanations for these findings include a change in pesticide usage in the area, control groups unintentionally receiving the intervention, or the intervention being inappropriate for the target audience. 

In another study, Bradman et al. (2009) recruited strawberry workers in the Salinas Valley of California and divided them into intervention and control groups at the workplace [54]. Six different intervention strategies were implemented to the intervention group: lightweight coveralls to wear over clothing and a weekly professional laundry servicing for said coveralls, disposable gloves, containers for storing work shoes and work clothing, warm water and soap to motivate frequent washing of hands, and five in-field educational sessions. The use of gloves during the workday seemed to be the most useful intervention strategy; the concentration of malathion on hands at the end of the workday as well as urinary pesticide metabolite concentrations were significantly lower among workers who wore gloves than those who did not. Similarly, malathion was measured on clothing but not on skin, which suggests that clothing is an effective barrier and reinforces the practice of keeping work clothes at the workplace in order to reduce take-home transport of pesticides. 

Salvatore et al. (2015) recruited women in an agricultural community in Salinas, California that were either the spouse/partner of a farmworker or a farmworker themselves and had a child at home aged four years or younger that was walking [20]. Eligible families were randomly assigned to intervention and control groups. The interventions were based on the social-cognitive theory and the health belief model and consisted of educational sessions on how to reduce take-home exposures. The intervention was delivered by trained community health workers in a one-on-one setting in the participant’s home. Children in the intervention group had a decrease in DAP metabolite concentrations post-intervention, but the difference was not significant. However, floor wipes taken from the intervention group had a decrease for only one of the measured pesticides. The authors acknowledged that upstream interventions in the workplace (e.g., workplace policies and controls to reduce workers’ exposure to pesticides and probability of transporting residues home such as providing laundering services) would be more effective than an in-home intervention. Finally, in a recent study conducted by Griffith et al. (2018) in the state of Washington, USA, a community-based participatory intervention was developed to reduce the exposure to pesticides exclusively via the take-home pathway. The intervention was based on several campaigns that included festivals and health fairs leaded by community health workers *(promotoras)* with the objective to communicate strategies to protect farm workers’ families from pesticides such as washing adult clothes separately of children’s clothes. To asses this intervention, urine and dust samples were collected before and after the intervention. After the intervention, the children’s exposure to OPs were significantly (*p* < 0.001) reduced with a 2.7-fold decrease from baseline measurements to the post-intervention measurement [37]. This intervention provides an effective process for disrupting the take-home pathway and reinforces how interventions can positively impact the reduction of pesticide exposure. 

### 3.8. Seasonality

Only three of the reviewed studies assessed the seasonal differences in urinary pesticide metabolites among children [41,49,55]. Fiedler et al. (2015) showed that seasonality plays an important role in pesticide exposure by comparing urinary TCPy levels in 24 children from an exposure group (rice farming community) and 29 from a control group (aquaculture shrimp community) [55]. TCPy concentrations were significantly higher during the low-pesticide-use season than the high-pesticide-use season for the exposure group (GM = 9.59 μg/g creatinine vs. GM = 6.06 μg/g creatinine). This difference was also observed for the control group, where TCPy concentrations were significantly higher during the low pesticide use season (GM = 4.31 μg/g vs. GM = 2.84 μg/g of creatinine) [55]. Household use of pesticides during the low-use season may have been attributed to the high urinary TCPy concentrations amongst the participants. In another study, Atrazine concentrations in household dust were higher during the planting season (April–June) (GM = 422 ng/g) than the non-planting season (November–December) (GM = 33 ng/g) season [49]. These studies found that OP metabolites in the urine or/and dust were high during the summer season, which corresponds to the pesticide application season for most regions. Additionally, during different agricultural seasons, Tamaro et al. (2018) evaluated pesticide exposure by collecting dust samples from 119 households and 270 vehicles, and urine samples from 171 adults and children living in a rural community of the state of Washington [41]. The study found that parental occupation can significantly increase the concentration of certain OPs in house dust and children’s urinary OP metabolite concentrations depending on the type of OP and agricultural season such as thinning and harvest [41]. 

### 3.9. Age and Gender as an Exposure Factor

Four studies assessed pesticide urinary metabolite levels differences based on age and/or gender. Rohitrattana et al. (2014) reported that creatinine-corrected total DAP (∑DAP) concentrations (rho = −0.31, *p* = 0.02) and TCPy concentrations (rho = −0.29, *p* = 0.03) were inversely correlated with age [40]. In a longitudinal cohort study of 400 children ages 6, 12, and 24 months in California, DMAP urinary metabolite concentrations were three times and two times higher at 24 months and 12 months compared to the children’s 6-month DMAP urinary metabolite concentrations [48]. Only one study evaluated the acetylcholinesterase (AChE) activity in relation to age and determined that each year of life was associated with an AChE increase of 0.05 U/mL [43]. With respect to gender, Panuwet et al. (2009) found that non-specific urinary metabolite concentrations for parathion were higher among males compared to females [38]. 

## 4. Discussion

A majority of the reviewed studies provided significant data on how farmworkers and their families are exposed to pesticides via the take-home pathway. Of the 39 articles found in this systematic review, only 9 articles have been previously summarized by Hyland and Laribi (2017) and 8 articles have been identified by Deziel et al. (2015) [11,12]. Thus, we have found 24 additional articles published in the last decade, from various locations globally, related to pesticide take-home pathway that were not considered in previous reviews. When compared to the previous literature review on the pesticide take-home pathway by Coronado et al. (2011b), there seems to be an increase in the number of studies using urinary biomarkers within the last decade, but a fewer number of studies that analyzed pesticides in dust and/or air [6]. In addition, blood measurements were considered for this systematic review and not in the Coronado et al. (2011b) review. In comparison to the previous literature reviews conducted on this topic, there seems to be a growing international interest in investigating the take-home pesticide exposure pathway. Our review indicates that research involving the pesticide take-home pathway is increasing outside of the U.S. (*n* = 14, 36%) in comparison to a fewer number of articles (*n* = 5, 16%) reviewed before the year 2008 by Coronado et al. (2011b). Similarly, when compared to other recent literature reviews, only one of the articles summarized by Hyland and Laribi (2017) was outside of the U.S, while Deziel et al. (2015) did not review any articles outside the U.S. In our review, we found that many of the studies conducted in developing countries reported higher pesticide biomarker concentrations in participants compared to those in the U.S., providing some evidence that more international policies and guidance to reduce pesticide exposure may be needed. While developed countries have restricted or banned certain pesticides, the effects on the developing world are unknown. It is possible that some of these pesticides are being used at an increased rate in developing countries. For example, the OP chlorpyrifos is widely used in developing countries due to its price and accessibility, while European countries and the U.S. have established strict regulations towards use of this pesticide because of its known toxicity. Thus, there needs to be more coordinated efforts at the international level, so these exposures are not disproportionately shifted to countries with less resources. Within the U.S., our review showed that there is a growing body of literature coming from California when compared to the studies reviewed from 1990 to 2008 by Coronado et al. (2011b). Also, there has been a considerable increment in the number of studies that analyzed the pesticide exposure pathways for pyrethroids (*n* = 11, 28%) in comparison to the Coronado et al. (2011b) review (*n* = 1, 3%), which may be explained by the high usage of this pesticide for household and agricultural purposes ubiquitously. On the other hand, even though glyphosate is more commonly used around the globe, our review only found one study (3%) exploring this herbicide in relation to the take-home pathway. 

Through this systematic review, it is clear that there continues to be growing evidence for the importance of the take-home exposure pathway. Another important factor to consider is household proximity to farms, since exposure to pesticides may be attributed to pesticide drifts from farm fields. Residing in or near an agricultural community with higher pesticide usage was associated with increases in markers of exposure to pesticides, as detailed in several of the reviewed studies [14,28,40]. These studies found that pesticide urine concentrations in children were not only significantly associated with proximity to farms, but also to being with the parent on the farm while they were working, playing on the farm, and presence of visible dirt accumulated on the child’s body [40]. 

When using urinary metabolites as biomarkers of exposure, it is important to consider the variability of metabolite concentrations within individuals. Griffith et al. (2011) conducted a longitudinal assessment over a 21-month period that evaluated urinary DAP concentrations in 44 children living in an agricultural community of Washington state [36]. This longitudinal study found that urinary OP pesticide metabolite concentrations varied more than three times for day-to-day measurements in a single child compared to differences between children, suggesting that individual factors contribute significantly to OP pesticide exposure [36]. Some of the factors that can influence individual temporal variability include pesticide application schedules, child’s household proximity to farm, children’s behaviors such as frequency of play in the nearby fields, and children’s mouthing and dermal contact behaviors [34]. Thus, it is challenging to distinguish between proximity to farms and the para-occupational or take-home pathway of exposure. Most of the reviewed studies measuring pesticide metabolites in urine suggested that proximity to farms and having a family member working in agriculture contributes to the risk of exposure to pesticides in homes, but none of these studies were able to determine if the para-occupational exposure pathway was more relevant than proximity to farms. Other pesticide exposure pathways such as residential application of pesticides and dietary intake also need to be considered when evaluating the relative contribution of the take-home exposure pathway on pesticides. 

This review also shows that regardless of the pathway of exposure, the pesticide application season in nearby farms should be considered when assessing the levels of exposure to pesticides in households. Also, although age and gender seem to be important factors to consider when evaluating pesticide exposure, there were only four studies that included these factors in relation to the take-home pathway as a main contributor to their exposure, so no conclusion can be made if age and gender are associated with increased exposure via this pathway and further research is needed. With respect to the health outcomes associated with pesticide exposure via the take-home exposure pathway, only four studies focused on the health effects, and many had varying areas of focus in their respective studies. Suarez-Lopez et al. (2013) found that systolic blood pressure was decreased in children living with flower workers [22]. Sutris et al. (2016) found a significant association between frequency of apple consumption, pesticide detection in urine, living in the area, and age with DNA damage/alteration in children [17]. Other studies have also reported associations between pesticide exposures and neurodevelopment in infants and young children. In Butler-Dawson et al. (2016), neurocognitive deficiencies, specifically decreases in divided attention and Purdue pegboard tests were found for children who lived in agricultural homes compared to those who lived in non-agricultural homes [56]. A study that compared neurobehavioral effects in children living in a rice farm community and children living in an aquaculture community found no significant adverse neurobehavioral differences after controlling for several pesticide metabolites (OPs and pyrethroids), children’s ages, and housing structures [55]. However, researchers in this study were not able to assess chronic exposure to pesticides and had a relatively small sample size. Hence, future studies should investigate the possible health outcomes associated to pesticide exposure via take-home pathway.

Additionally, this systematic review supports the idea that common home and work practices can increase or reduce the pesticide exposure. For example, the reduction of pesticide levels when work shoes were not worn in homes or the increase in urinary metabolite levels in homes that frequently applied hand sanitizers [18,49,50,52]. The latter example may be explained by the chemical makeup of hand sanitizers, as alcohols are known to increase penetration of other chemicals through the stratum corneum, it is likely that these hand sanitizers also increase the dermal absorption of pesticides [57]. Many studies examined individual behavior and whether workplace safety behaviors and tasks predict household dust or biomarker concentrations in family members of occupationally exposed workers. Workplace practices such as mixing/loading pesticides and cleaning spray equipment are associated with greater inhibition of cholinesterase activity (biomarker of exposure), while factors such as wearing a full-face respirator, wearing chemical-resistant footwear, and storing personal protective equipment in a locker at work are protective against inhibition of cholinesterase activity [58]. Therefore, our systematic literature review presented several studies that implemented and evaluated interventions at the community and workplace level with the objective of reducing the levels of pesticides transported via the take-home pathway. Most of the interventions on interrupting the take-home pathway presented in this review succeeded in reducing the exposure to pesticides, again reinforcing the take-home pathway as an important route to consider in exposure assessment. 

### Limitations

We have identified several study limitations that impact the ability to evaluate the relative contribution of the take-home pathway to exposure and potential health impacts. Perhaps the greatest limitation is that for many of the studies it was difficult to determine the independent contributions of the take-home pathway in comparison to proximity to farms and residential pesticide use [17,31,40]. This was, in part, because many of the rural communities where these studies were conducted in households that had at least one agricultural worker (suggesting occupational exposure), the homes were located in close proximity to farms where pesticides were sprayed and participants oftentimes reported frequent residential pesticide applications. The frequent co-occurrence of these factors makes it difficult to disentangle the independent effects of the take-home exposure pathway.

In addition, most of the reviewed studies relied solely only on human biomarkers while only 10 studies evaluated both environmental dust samples dust and biomarkers, specifically urine, at the same time. None of the environmental–biomarker combination studies reviewed used other biological matrices such as amniotic fluids, hair, or nails. Furthermore, all of the studies measured metabolites and not the parent compounds in urine and blood. Thus, as pointed out in the previous literature review, it is not clear what contribution of the metabolite levels are due to exposure to parent pesticide compounds or to exposure to their breakdown products in the environment [9]. Also, these biomarker levels reflect multiple exposure routes and pathways, as well as multiple parent pesticides if the biomarkers are non-specific like DAPs. While the use of biomarkers provides an integrated measure of exposure, it does not allow researchers to identify which specific pathway of exposure dominates. Thus, it is often difficult to distinguish the relative contributions of exposure pathways and parent pesticides, making it difficult to design effective intervention studies. Additional measurements such as environmental samples (e.g., air, dust, soil), dietary samples (e.g., food) or intake assessment instruments (e.g., food frequency questionnaire) may provide additional information and reduce exposure misclassification. For example, external measures such as air samples to could be used to better delineate exposure routes (inhalation) and pathways (pesticide drift). 

Another limitation is that the majority of the reviewed studies resulted from the collection of a single biological or/and environmental sample, rather than multiple samples per participant, which can provide more reliable and less variable findings. Most of the studies also relied on spot urine samples, which may also have more variability than 24-hour collection or first-morning void samples. There was lack of homogenous study designs and methods, which increases the variability and uncertainty of the results, and also made it difficult to compare across studies, geographic regions, and exposure pathways. Another limitation was small sample size, as the majority of the studies had sample sizes of fewer than 200 participants with many of the studies reporting less than 50 participants. 

Publication bias may have also biased our analysis, as researchers are less likely to publish and report null associations or negative findings. However, we found four studies with no evidence supporting the exposure via take-home pathway [18,19,20,21]. An important limitation in our systematic review is that articles written in a language other than English were excluded from this analysis. Thus, we may have missed reviewing additional articles from international regions. In our review, some of the highest pesticide levels were reported from geographic regions that do not primarily speak English.

## 5. Conclusions

This systematic literature review provides the latest evidence that the take-home exposure pathway is an important contributor to overall residential pesticide exposures. However, this pathway should not be considered solitarily. Other pathways such as pesticide drift, indoor-residential applications, and dietary intake of pesticide residues in food can be equally important. We found that studies investigating the pesticide take-home pathway should also consider factors such as age, gender, and seasonality in their research plans. Therefore, a more comprehensive exposure assessment approach is necessary to better understand both aggregate and cumulative exposures to pesticides so more effective interventions can be created. Only a few studies considered multiple exposure pathways. These few studies focused on residential proximity to farms, occupational status, dietary-intake, and the take-home pathway. However, most of the studies only utilized biomarkers to assess exposure, which may relate more to dietary exposures and may underestimate the effect of dermal and inhalation exposure. Consequently, there is a need for more multimedia monitoring to help identify sources and pathways of exposure. Additionally, it is important to recognize that there is an increased number of studies evaluating the exposure to pyrethroids when compared to previous reviews. Although the majority of the reviewed studies focused on analyzing exposures to OPs and pyrethroids, there is a lack of research on other pesticides including glyphosate, which is currently one of the most widely used pesticides worldwide, and neonicotinoids. In comparison to previous literature reviews, there seems to be a growing international interest in researching the take-home pesticide exposure pathway within the last decade. Finally, as presented in some studies on interventions of individual behavior to reduce pesticide exposure, we found that most community-based interventions that focused on interrupting/blocking the take-home pathway were able to reduce pesticide exposure in households.

## Figures and Tables

**Table 1:** Literature review summary on take-home pathway of pesticide exposure - blood biomarkers.

Author (Date)	Location	Sample Size	Pesticide(s) Measurement	Sample Type	Main Findings
Ali et al. (2013)	Pakistan	34 mothers (23–51 years), 34 children (3–10 years), 17 general group (13–65 years) living in rural and agricultural areas	OC metabolites	Blood	The ∑DDTs metabolites in serum were significantly higher in rural children (median: 535 ng/g) than urban children (median: 250 ng/g). A significant correlation between rural children and their mothers for ∑DDTs (*r* = 0.461, *p* = 0.031) * was found, but no correlation in urban settings.
Gonzalez-Alzaga et al. (2018)	Spain	133 children living in agricultural community	OC metabolites	Blood	There were no associations of OC metabolites and children living near crops or greenhouses, family income, parental education, mother and/or father working in agriculture, or occupational exposure to chemicals of mother or father.
Huen et al. (2012)	California	526 Mothers living near farmland. Blood (n = 234), umbilical cord (n = 256) and urine samples from 221 mothers and 244 children.	OP metabolites PON1 enzyme	Mothers blood, umbilical cord blood and urine	Chlorpyrifos was detected in 71% and 88% of blood and umbilical cord samples. High maternal PON1 levels are related to decreased detection of diazinon and chlorpyrifos (OR: 0.75, and 0.56 and 0.75, respectively). Blood OP metabolites were similar in mothers and newborns and slightly higher than those reported in other populations.
Suarez-Lopez et al. (2012)	Ecuador	277 rural children (4–9 years old) in agricultural communities cohabitating with flower workers vs. non-flower workers	AChE activity	Blood	Cohabitation with a flower worker was related to lower AChE activity in children. This supports the hypothesis that the amount of take-home pesticides from flower workers suffices to decrease AChE activity, as lower AChE activity was associated with higher pesticide exposure.
Suarez Lopez et al. (2013)	Ecuador	271 children (4–9 years old), approximately half cohabitated with flower workers	AChE activity and blood pressure	Blood	Children living with flower workers had lower systolic blood pressure (−1.72 mmHg; 95% CI: −3.53, 0.08) than other children not living with farmworkers.

* Spearman rank correlation. Abbreviations: DDT, dichlorodiphenyltrichloroethane; PON1, Serum paraoxonase/arylesterase 1; AChE, acetocholinesterase; BuChE, Butyrylcholinesterase; OC: Organochlorine pesticides.

**Table 2:** Literature review summary on take-home pathway of pesticide exposure—urine concentrations.

Author (Date)	Location	Sample Size	Pesticide(s) Measurement	Sample Type	Main Findings
Babina et al. (2012)	Australia	340 Children (2.5–6 years old)	OP and pyrethroid metabolites	Urine	Higher urinary metabolites for OPs and pyrethroids were found in rural and periurban children compared to urban children whose parents did not report occupational contact with pesticides. Significantly higher levels of pyrethroids in children living within 50 m from an agricultural area.
Bradman et al. (2011)	California	416 children at multiple time points (6 months, 12 months, and 24 months)	OPs metabolites	Urine	DMAP levels were significantly higher in children living in households with at least one agricultural worker vs. none (21 vs. 11 nmol/L (*p* < 0.01)). There was a significant difference if their mother was an agricultural worker vs. not (29 vs. 16 nmol/L (*p* < 0.01)). No relation found of farmworker at home nor farmworkers wearing work clothes inside the house.
Fiedler et al. (2015)	Thailand	24 children agriculture and 29 children aquaculture community (6–8 years old)	OP and pyrethroid metabolites	Urine	OPs and PYR metabolites were significantly (*p* < 0.005) higher in children living in agricultural areas compared to children from an aquaculture community. Proving that proximity to pesticide applied areas and take-home pathway may increment children exposure to pesticides.
Griffith et al. (2011)	Washington State	44 children in agricultural community (2–5 years old)	OP metabolites	Urine	OP exposure appeared to vary more than 3 times from day-to-day than from child-to-child showing that individual variability needs to be considered. Proximity to farms, food intake, and take-home pathway may influence on variability.
Hanchenlaksh et al. (2011)	Thailand	16 farmworker families	OP metabolites	Urine	Farmers’ urinary DAP metabolites were not correlated with those of their children (GM: 7.6 μg/g) or spouses (GM: 13.0 μg/g). The main route of exposure seemed to be from farmer to family members home. Farmer showering at work was an important determinant to reduce exposure.
Kongtip et al. (2014)	Thailand	Women during pregnancy (n = 86), delivery (n = 67) and 2 months postpartum (n = 51)	OP metabolites	Urine	The main factors that influenced the urinary metabolite concentrations during pregnancy were frequency of agricultural field visits during the first and second trimesters of pregnancy, and subjects’ occupations.
Mesnage et al. (2012)	France	Case study: Male farmer, wife, and three children	Herbicide (Glyphosate)	Urine	Glyphosate was detected in the urine of the male farmer, but was not detected in the farmer’s wife or two children’s urine. Only one child had detectable levels of glyphosate in urine.
Panuwet et al. (2009)	Thailand	207 children (12–13 years old); grouped by parents’ (farmers and non-farmers)	OP, pyrethroid, and herbicide metabolites	Urine	Children of farmers had significantly higher (*p* < 0.05) levels of pyrethroid metabolites than children of non-farmers.
Raymer et al. (2014)	North Carolina	361 men living in employer-provided farm worker housing	OP, pyrethroid, and herbicide metabolites	Urine	The metabolite levels of 2-Isopropyl-4-methyl-6-hydroxypyrimidine were significantly increased when workers reported changing their clothes in sleeping room (*p* = 0.017), and when they reported storing their clothes (*p* = 0.031) and shoes (*p* = 0.041) in the sleeping room.
Rohitrattana et al. (2014)	Thailand	24 children from rice-growing community; 29 children in aquaculture community (both groups 6–8 years old)	OP metabolites	Urine	Increasing TCPY levels found in children were significantly related to being with a parent who worked at a farm (*p* = 0.02) and the levels were higher than children who lived in aquaculture communities. In general, OP metabolites were associated with farm activities, house environments, and behaviors.
Sutris et al. (2016)	Malaysia	180 children (7–12 years)	OP metabolites	Urine	Children with farmworker parents had 3 times higher risk of DAPs detection levels than children with non-farmworker parents.
Wu et al. (2013)	China	513 infants (1 year old)	Pyrethroid metabolites	Urine	Children whose fathers worked in agriculture had twice the amount of PYR metabolite concentration than children whose parents were not farmers (GM: 0.90 µg/L and 0.47 µg/L). No correlation was found between urinary metabolites and maternal occupation. Home ventilation was associated with lower urinary metabolites concentrations.

Abbreviations: OP, organophosphates; DAP, dialkylphosphate; PYR, pyrethroids; DMAP, dimethyl alkylphosphate; 3-PBA, 3-phenoxybenzoic acid; PNP, paranitrophenol; TCPY, 3,5,6-trichloro-2-pyridinol; GM, geometric mean.

**Table 3:** Literature review summary on take-home pathway of pesticide exposure—dust and urine sample concentrations.

Author (date)	Location	Sample Size	Pesticide(s) Measurement	Sample Type	Main Findings
Bradman et al. (2009)	California	44 strawberry harvesters: 15 in control group and 29 in the intervention group	OP residues and metabolites	Urine, hand rinse, clothing patch	Educational intervention and providing PPE resulted in lower MDA (on hands and in urine) among workers who wore gloves; wearing gloves and taking off clothes may reduce transport of pesticides to homes. MDA was detected on clothing (median = 0.13 μg/cm^2^), and not on skin. Also, MDA on hands was significantly lower (*p* < 0.001) among workers who wore gloves compared to those who did not (median = 8.2 μg/pair and 777.2 μg/pair, respectively).
Coronado et al. (2011a)	Washington State	100 farmworker and 100 non-farmworker families (children ages 2–6 years old)	OP residues and metabolites	Urine, Dust	Farmworker (FW) households had higher levels of dust OP metabolites than non-FW’ households. DMTP concentrations in urine were higher in FW than non-FW families (GM: 71 μg/L and 6 μg/L, respectively). FW children had higher levels of OP metabolites in urine than non-FW children (GM: 17 μg/L and 8 μg/L, respectively). A 20% increase in DMTP concentration was observed per mile closer to farmland.
Coronado et al. (2012)	Washington State	95 orchard workers and 94 children (2–6 years old)	OP residues and metabolites	Urine, Dust	No significant associations between work practices and levels of AZM in house dust. No significant differences in AZM urinary metabolite in children living with a farmworkers, stratified by work activities. AZM levels in house dust was not associated with workplace practices. Workers who used hand sanitizer had higher urinary concentrations of DMTP, as did children who attended daycare.
Griffith et al. (2018)	Washington State	Year 1: 197 adults and 186 children. Year 4: 187 adults and 172 children. Urine samples: 383 in pre and 359 in post intervention period.	OP residues and urine metabolites	Urine, Dust	The intervention significantly reduced children’s exposure to OPs: The child/adult pesticide ratio for the intervention group had a 2.7-fold decrease from the baseline value of 0.32 to the post-intervention value of 0.12 (*p* < 0.001). The community intervention program of disrupting the OP take-home pathway had a significant (*p* < 0.001) reduction in children’s urinary DMTP levels.
Quirós-Alcalá et al. (2012)	California	20 children from urban families (3–6 years); 20 children from rural families (3 to 6 years)	OP residues and metabolite	Urine, Dust	DEP was the most frequently detected OP in urine (>60%). DEP dust concentrations were not significantly different between the two communities. OP concentrations in dust were not a significant source of DAPs in urine.
Salvatore et al. (2015)	California	116 families (children urine: 106; floor wipes: 103)	OP and pyrethroid residues and metabolites	Urine, Dust	No significant associations with urinary DAP levels and the number of household members working in agriculture. Educational intervention at home was significantly associated with a 37% decrease in pyrtehroids floor wipe levels in homes, but not OPs.
Tamaro et al. (2018)	Washington State	Households: 119 House dust: 498 Vehicle dust: 270 Urine samples: 171 adults and 170 children	OP residues and metabolites	Urine, Dust	The parental occupation influenced the concentration of dimethyl OPs in house dust in the thinning season. During the harvest season, the children’s concentration of urinary DEP and DETP was influenced by the parent occupation as well. Diethyl OPs in house dust in any season was not affected by parental occupation.
Trunnelle et al. (2014)	California	105 women and 103 children (2–8 years old)	Pyrethroid metabolites	Urine, Dust	Urinary metabolites were higher than in U.S. general population. Children had higher metabolite levels than their mothers. A significant positive association was found between outdoor cypermethrin levels and the levels found in indoor dust samples. Poor housing conditions and the urinary metabolite levels were significantly correlated (rs = 0.28, *p* = 0.0450).
Thompson et al. (2008)	Washington State	24 communities: 205 adults and 204 children (1 year old), 202 adults and 204 children (4 years old).	OP residues and metabolites	Urine, Dust	Community-wide intervention on take home pathway had no effect on pesticide concentrations in urine and dustvehicles and house dust.
Thompson et al. (2014)	Washington State	100 farmworker and 100 non- farmworker families	OP residues and metabolites	Urine, Dust	The dust in the vehicles and houses of FW had higher OP residue concentrations than in non-FW vehicles and houses. FW families had significantly higher urinary DMPTP metabolite concentrations than non-FW families during application season (mean DMTP: 16.5 μg/L for FW children vs. 7.5 μg/L for non-FW children), but no significant differences were found during off season.

Abbreviations: OPs, organophosphates; DAP, dialkylphosphate, DMTP, dimethylthiophosphate; MDA, malathion; DEP, diethylphosphate; AZM, Azinphos-methyl.

**Table 4:** Literature review summary on take-home pathway of pesticide exposure—dust and air sample concentrations.

Author (date)	Location	Sample Size	Pesticide(s) Measurement	Sample Type	Main Findings
Arcury et al. (2014)	North Carolina	176 migrant farmworker houses	OP and pyrethroid residues	Dust	The concentration of OPs chlorpyrifos (GM: 0.11 μg/m^2^) and malathion (GM 0.12 μ/m^2^) found in the houses were not associated with camp characteristics. The concentrations of pyrethroids present in the migrant farmworker houses were associated with specific camp characteristics.
Butler-Dawson et al. (2016)	Pacific Northwest, U.S.	155 farmworker households and 60 non-farmworkers households (children 5–12 years old)	OP residues	Dust	Children in agricultural households had higher risk for exposure to OPs as there were significantly (*p* < 0.01) higher amounts found in their house dust compared to non-agricultural children (e.g., chlorpyrifos median: 18.0 ng/g and 7.0 ng/g, respectively)
Butler-Dawson et al. (2018)	Pacific Northwest, U.S.	278 households in an agricultural area	OP residues	Dust	The odds of having high azinphos-methyl concentrations were 6.25 times more likely in homes with two or more agricultural persons compared to homes having only one agricultural worker (OR: 3.14, *p* < 0.10 and OR: 4.07, *p* < 0.10, respectively).
Fenske et al. (2013)	Washington state	46 farmworkers (handlers: 16 thinners: 15 reference: 15)	OP residues	Dust	Differences were found across worker groups for availability of laundry facilities, work boot storage, frequency of hand washing, commuter vehicle use, parking location, and safety training. Vehicle and house dust significantly correlated with each other. Interventions need to be closer to contamination source to reduce take home pesticide exposure.
Golla et al. (2012)	Iowa	32 households in agricultural community; 256 dust samples	Herbicide (Atrazine residues)	Dust	Atrazine concentrations had significantly (*p* < 0.05) decreased (GM: from 422 ng/g to 33 ng/g) several months after application in fields, but it was still detected, indicating potential for long-term exposure and continued transport into homes. Modifications in work practices and personal hygiene did not show a reduction into home atrazine levels.
Gunier et al. (2011)	California	89 households in agricultural community	OP residues	Dust	Chlorpyrifos concentrations in carpet was higher in farmworkers households than non-farmworker household (median: 100 ng/g and 37 ng/g for, respectively). Living within 1250 m of agricultural land significantly increased the dust concentrations of pesticides inside homes.
Harnly et al. (2009)	California	168 households in agricultural community; 504 samples of households near farms	OP, pyrethroid herbicide, and fungicide residues	Dust	OP concentrations in dust significantly (*p* < 0.01) increased as pesticide applications in nearby farms increased (except for diazinon and chlorpyrifos). Pesticide concentration in dust was significantly(*p* < 0.05) associated with storing of shoes inside farmworkers’ homes.
Plascak et al. (2018)	Washington state	91 households; 418 dust samples	OP residues	Dust	The GM (95% CI) of dimethyl OPs residues in homes with none, one, or at least two residents working in agriculture was 0.07 nmole/g (0.02–0.31), 0.12 nmole/g (0.03–0.56), and 0.35 nmole/g (0.07–1.75), respectively. The pesticide house dust concentrations among homes with at least two agriculture workers was approximately 400% higher than among homes without any agriculture workers, regardless of orchard density.
Norkaew et al. (2013)	Thailand	108 households of farming and non-farming families	OP residues	Air, dust	Houses of farmworker families had higher levels of pesticide residues than non-farmworker families; however, the chlorpyrifos mean concentration for air and dust samples were not significantly different between farmworker and non-farmworker families.
Quirós-Alcalá et al. (2011)	California	13 urban houses and 15 farmworker homes	OP, pyrethroid, herbicide, and fungicide residues	Dust	Analytes detected in both urban and farmworker homes had no differences in concentrations or loadings between the locations. Chlorthal-dimethyl was detected solely in farmworker homes. Diazinon and chlorpyrifos concentrations in urban homes were 40–80% lower than the concentrations seen in FW homes.
Smith et al. (2016)	Washington state	100 farmworker (FW) households; 100 non-farmworker (non-FW) households	OP residues	Dust	Pesticide dust concentrations were higher in FW households than non-FW households (e.g., Chlorpyrifos dust concentrations were 9.8 times higher, *p* < 0.05, in FW than non-FW houses).
Trunnelle et al. (2013)	California	55 farmworker households	Pyrethroid residues	Dust	Cis- and trans-permethrin had the highest detection frequency (67%) (med: 244 and 172 ng/g). Deltamethrin, esfenvalerate, and permethrin were detected more frequently in this study than other studies. A positive association was found between cypermethrin outside and cypermethrin dust concentrations inside households (rs = 0.28, *p* = 0.0450) *

* Spearman rank correlation. Abbreviations: GM, geometric mean; CI, confidence interval; OPs, organophosphates; DAP, dialkylphosphate, DMTP, dimethylthiophosphate; MDA, malathion; DEP, diethylphosphate.
